# Supplementary material for: Identification of photoperiod-induced specific miRNAs in the adrenal glands of Sunite sheep (Ovis aries)
Source: Front Vet Sci. 2022 Jul 22;9:888207. doi: 10.3389/fvets.2022.888207 (PMC9354845; doi:10.3389/fvets.2022.888207)
Supplement: Supplementary file 1 [file Data_Sheet_1.ZIP › Supplementary file/miRDeep2 file.zip!/miRDeep2 file/Novel_Structure/SPLP42A1/Novel_558.pdf]

| | | |
|---|---|---|
| Provisional ID | : | NC_019464.2_12965 |
| Score total | : | 248.5 |
| Score for star read(s) | : | 3.9 |
| Score for read counts | : | 240.8 |
| Score for mfe | : | 2.2 |
| Score for randfold | : | 1.6 |
| Score for cons. seed | : | |
| Total read count | : | 484 |
| Mature read count | : | 483 |
| Loop read count | : | 0 |
| Star read count | : | 1 |

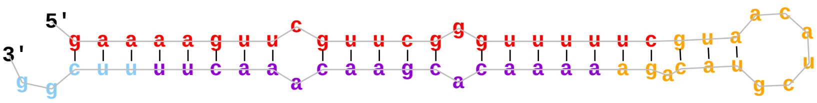


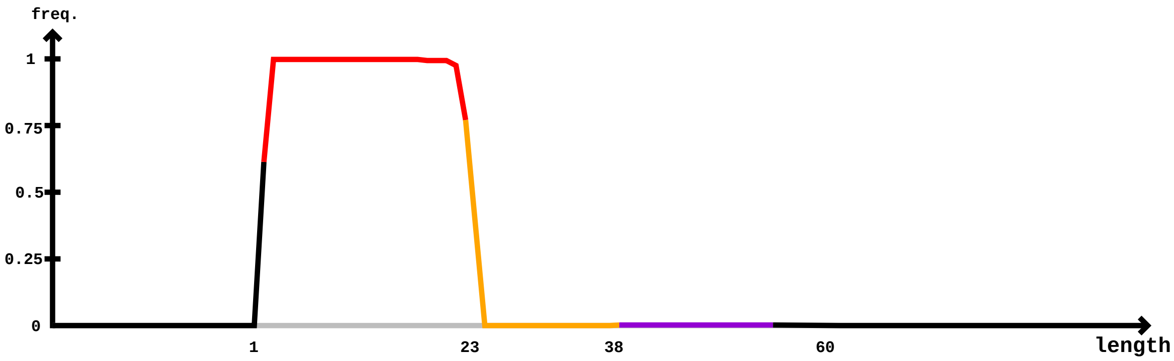


```
                            Mature                                     Star
5'- auuaucauauuggguuggcugaaaaguucguucggguuuuucguaacaucguacagaaaaacacgaacaaacuuuucggccaacccaguauuucauucaucugcguuuuccu   -3'    obs
    auuaucauauuggguuggcugaaaaguucguucggguuuuucguaacaucguacagaaaaacacgaacaaacuuuucggccaacccaguauuucauucaucugcguuuuccu          exp
    ......((((((((((((((((((((((.(((((.((((((((((......))).))))))).))))).))))))))))))))))))))))).....................   reads   mm   sample
    ....................gaaaaguucguucgggu...........................................................................   2       0    seq
    ....................Aaaaaguucguucggguuuuuc......................................................................   2       1    seq
    ....................gaaaaguucguucggguuuuuc......................................................................   293     0    seq
    .....................aaaaguucguucggguuuC........................................................................   2       1    seq
    .....................aaaaguucguucggguuuu........................................................................   7       0    seq
    .....................aaaaguucguucggguuuuu.......................................................................   99      0    seq
    .....................aaaaguucguucggguuuuuc......................................................................   66      0    seq
    .....................aaaaguucguuUggguuuuucg.....................................................................   7       1    seq
    .....................aaaaguucguucggguuuuucU.....................................................................   5       1    seq
    .........................................................aaaacacgaacaaacuu......................................   1       0    seq
```
